# Public and private fundraising as a tool for professional development: What is fundraising?

**DOI:** 10.1590/acb388123

**Published:** 2023-12-01

**Authors:** Everson Luiz de Almeida Artifon, Mariana Gonçalves Magon, Marcio Roberto Facanali, Edna Frasson de Souza Montero

**Affiliations:** 1 Universidade de São Paulo – Faculdade de Medicina – São Paulo (SP) – Brazil.; 2 Sorocaba Administrative District – Sorocaba (SP) – Brazil.

Targeted and qualified fundraising or mobilization is a necessary way of implementing projects, in support of their main purpose, public policies and investments that are generally not possible to execute only with own resources, whether in governmental or non-governmental, public or private organizations, regardless of the source or method to generate them^1^^,^ 2. Resources can be government, own or transferred.

Among the great challenges of organizations, the disparity between demands and available resources is exorbitant. In order for the results to be tangible and have an impact, it is essential to be attentive and agile in attracting resources.

Knowing one’s own budget and that of other entities that may become a source of resources, understanding the ecosystem that surrounds it is extremely timely for the search for better investments.

Although there is no regulation for the profession of fundraiser, being created according to the needs of the entities, it is important to notice that the search for complementary resources is a method of identifying opportunities and partners to explore and test new business initiatives, enabling the best use, optimizing efforts, and obtaining better results.

Although the term *fundraising* points exclusively to the financial understanding, the concept is much broader and also covers those resources related to technical, technological, material, immaterial, and human capacity, both for creation and implementation^2^^,^ 3.

Actions involving these activities can be applied to collaborate specifically with an institution (whether public or private), municipalities, states and/or priority projects and programs^4^.

The scarcity of capital directly affects entities, generating the most diverse limitations, from the provision of basic services, infrastructure, updates, and the implementation of continuous improvements through research and academic studies^4^^,^ 5.

An example is the current crisis caused by the coronavirus, which has required a large volume of investments in health in a short period of time. In order to reduce the health crisis, public resources were redirected to the Brazilian Unified Health System (SUS) in the three spheres of government. Although the private sector has supported the SUS, it has not received the same opportunities and benefits.

The private sector suddenly had to cope with an increase in demand for COVID-19 care, a rise in the price of supplies, and the suspension of elective surgeries and examinations, sharply reducing the revenues of private hospitals.

In the third sector, the pandemic has affected the budget of entities. According to Luis Donadio, coordinator of Fundraising Office of Fundação Oswaldo Cruz (Fiocruz), with the COVID-19 pandemic, it was found that of the R$ 7 billion received by Fiocruz during the pandemic, from the various entities of society, 80% had been operated by public institutions^6^^,^ 7.

The pandemic itself was one of the main recent factors that demonstrated how fragile and limited the budgets of institutions and governments are, sensitizing them on the importance of structuring areas and sectors focused on fundraising activities^8^^,^ 9.

Other challenges that make resources more constrained are the economic, legal, and political scenarios in Brazil.

However, it is not enough just to raise funds, with the structuring of areas and sectors focused on these actions. It is essential to qualify the human resources involved, training the teams to prepare plans based on priorities and feasibility of implementation, considering all the perspectives that the project requires, monitoring the technical and financial execution until completion, aiming at efficient and effective management.

When drafting, the adequacy of real targets, objective and up-to-date value estimates, the assessment of deadlines and their follow-up forms help to implement feasible projects.

Setting feasible targets, acapacity building, monitoring indicators, investing in institutional relationships, and monitoring project implementation are essential for achieving favorable results.

## Raising public funds

Aware of the competing demands on their own resources, external fundraising alternatives are sought.

There are several formats of resource transfers from public entities to other spheres, widely served in other areas as essential as health, briefly explained here only to add knowledge to the reader, since the focus of this work is the health area^10^^,^ 11:

- Constitutional transfers: the portions of resources collected by the Union and transferred according to the Federal Constitution;
- Legal transfers: regulated by representative laws that determine the qualification, application, and accountability. They may or may not be linked to a specific purpose. They are presented in automatic transfers, which consist of financial transfers without the use of covenants, adjustments, agreements, or contracts, occurring through deposit in a specific account of the beneficiary, used for the decentralization of resources in certain programs in the area of education;
- Direct transfer to citizen: provides monthly financial benefits to the target audience of the program;
- Voluntary transfers: for costing or investment, as cooperation, aid or financial assistance not related to constitutional or legal determinations.

The main financial provider for health care, public resources can be obtained by governmental and non-governmental (non-profit) entities.

Constitutional Amendment No. 29 of 2000 ensures the financing of public health actions and services, proposing that the three spheres of government annually contribute minimum resources from percentages of revenues. Expenses for public health actions and services are considered to be those with active personnel and other costing and capital expenses, related to finalistic and support programs, including administrative ones, which are aimed at actions and services of universal, equal and free access and which are in accordance with the objectives and goals of the health plans of each federative entity^11^.

Ordinance GM/MS No. 204 of January 29, 2007, regulates the financing and transfer of resources for health actions and services, monitoring and controlling^11^^,^ 12.

Currently, the distribution of public resources, mainly from the Union, is guided by the Federative Pact, established by the Federal Constitution of 1988. This pact determines the financial obligations, laws, collection of resources, fields of action and the definition of the competencies of each entity of the federation–Union, states, and municipalities.

The Constitution provides that each sphere of government must apply a minimum share of tax revenues to health, with 12% of revenues for states and 15% for municipalities.

The National Health Fund, established by Decree No. 64,867 of 1969, acts at the federal level as the financial manager of SUS resources, financing capital and current expenses, in accordance with the policies and programs managed by the final secretariats of the Ministry of Health, in addition to managing the transfers of these resources to states, municipalities and the Federal District^7^^,^ 8 ^,^ 10.

The budget allocations that make up the general budget of the Union, intended for the transfer of resources, are:

- Program Resources: programs previously listed by the grantor, available for public and private entities to register project proposals;
- Parliamentary Amendment Resources: these are resources granted to parliamentarians of the Legislative Branch, inserted in the General Budget of the Union, with a view to participating and influencing the distribution of the proposals of the Executive Branch. The distribution by the ministry occurs through current legislation and adapting to the technical character of execution^13^.

After the publication of Ordinance No. 3,992/2017, federal funds for public health actions and services began to be transferred in the fund-to-fund modality, through two blocks^13^^,^ 14:

- Public Health Actions and Services Costing Block: resources intended for public health actions and services already implemented and the functioning of the responsible bodies and establishments.
- Investment Block in the Public Health Services Network: resources for the structuring and expansion of public health actions and services.

**Table:** 

Final objects	
Capital - Investment	Costing - Current
Construction of a health unit	Health unit maintenance
Expansion of the health unit	Health unit reform
Acquisition of equipment	Human resources traing
Acquisition of permanent material	Studies and research

Source: Primer for Submission of Proposals to the Ministry of Health^11^.

The Investment classification (GND 4) is used for budget expenses with the execution of expansion works and new constructions or with the acquisition and installation of equipment and permanent material. The transfer occurs via fund to fund, agreement or transfer contract, taking into account the structuring of specialized health care units or the structuring of the primary health care services network^10^^,^ 12.

It is noteworthy that only the acquisition of equipment and permanent materials that are part of the National List of Equipment and Permanent Materials that can be financed for SUS is allowed^7^.

The classification of Other Current Expenses (GND 3) is used for budget expenses for the acquisition of consumables, renovations, training, in addition to other current expenses of the other nature groups. The transfer occurs via fund to fund, considering the temporary increment of the medium and high complexity ceiling or the temporary increment to the costing of primary health care services^10^.

Before sending the resource, it is recommended to consult the primary health care services and medium and high complexity ceilings of each state, the Federal District, and each municipality to be indicated as beneficiary. If the ceiling is already exceeded, it is not possible to receive new resources within the current year.

No counterpart is required from states, municipalities or the Federal District for transfers of resources to SUS, as well as for private non-profit entities that work in health and comply with art. 76 of Law No. 13,242/2015^13^^–^ 15.

The instruments for the transfer of resources from the Ministry of Health to the states and municipalities are carried out by:

- Fund-to-fund transfers;
- Agreements;
- Onlending contracts;
- Decentralized execution term;

Public consortia, created by Law No. 11.107/2005, establish the joining of two or more federation entities with legal personality to establish cooperation relations and the achievement of common objectives. Art. 9 of Interministerial Ordinance No. 127/2008 determines that public consortia have preference to voluntary transfers from bodies and entities of the federal public administration.

These transfers of resources from the Union to the other spheres of public administration or to private non-profit entities are carried out through the conclusion of instruments between the parties, for the execution of activities of reciprocal interest, highlighted in the general budget of the Union in budget appropriations for specific purposes.

Prioritizing the capture of public resources in health, according to the classifications already mentioned, the following describes in detail the transfer instruments.

## Health fund-to-fund transfers

Fund-to-fund transfers decentralize resources by dispensing with the formalization of agreements. Guided by Law No. 8,142/1990 and regulated by Decree No. 1,232/1994, they are transfers that are developed within the scope of the Brazilian SUS, through the National Health Fund, under the management of the Basic Operational Standard of the Brazilian SUS 01/1996^10^.

Intended to cover health actions and services that are implemented by the states, Federal District, and municipalities, these actions and services correspond to investments in the service network, outpatient and hospital coverage and other health actions.

The decentralization process was improved by the Ministry of Health in 2006, with the release of the Health Pact (Ordinance No. 399/2006). Thus, the transfers became part of six resource blocks: basic care (primary care), medium and high complexity care, health surveillance, pharmaceutical assistance, SUS management, and unregulated block^12^.

Transfers occur directly from the National Health Fund to state, Federal District, and municipal health funds. The application must be carried out as provided for in the health plan of the respective government sphere. It should reflect, at the end of each year:

- The link with the purpose of each work program of the general budget of the Union that gave rise to the transfer;
- The established in the health plan and in the annual health programming of each federative entity;
- The object and commitment agreed in the normative acts of the SUS.

When directing resources to municipalities, according to Law No. 8,142/1990, the municipality must meet the minimum criteria, which include the existence of a health fund, health council, health plan, management report, counterpart of resources in the respective budgets, and a commission for the elaboration of the career, positions and salaries plan, with a two-year deadline for its implementation. Failure to comply with these criteria will result in the State being responsible for administering the funds^9^.

With regard to accountability, Consolidation Ordinance No. 1/GM/MS of 2017 regulates the management report by consolidating the rules on the rights and duties of health users, the organization and functioning of the Brazilian SUS^14^^,^ 15.

This management report must be sent to the Ministry of Health annually and submitted to the respective health council for approval, proving the conformity in the application of the transferred resources (adequacy with the purpose of the budget action), and compliance with the agreed object.

## Covenants and on-lending contracts

Interministerial Ordinance 424, of December 30, 2016, establishes: Agreement is an instrument that regulates the transfer of financial resources from bodies or entities of the Federal Public Administration, direct or indirect, to bodies or entities of the State, District or Municipal Public Administration, direct or indirect, public consortia, or private non-profit entities, for the purpose of executing a project or activity of reciprocal interest and in mutual cooperation^5^^,^ 11.

Decree No. 6,170, of July 25, 2007, establishes that the on-lending contract is the administrative instrument in which the transfer of financial resources is carried out through a federal public financial institution or agent, as agent of the Union^6^^,^ 11.

The rules apply to agreements and, when applicable, to transfer contracts.

## Term of decentralized execution

The decentralized execution term is the decentralization of credit between bodies and / or entities that are part of the Union’s fiscal and social security budgets, to carry out actions of interest to the decentralizing budget unit and the object provided for in the work program, faithfully respecting the functional programmatic classification.

## Private fundraising

The health sector in Brazil has faced numerous difficulties, which has required an urgency on the part of institutions and organizations in diversifying to contribute resources to their projects. The professionalization of project management has become increasingly necessary and resolutive in view of the prospects and projections for the economic scenario in the country.

In order to finance investments, alternatives are emerging in the private sector that can help to complement public resources, which are currently insufficient for health maintenance.

Although the search for resources in the private sector provides a wider range of options, these follow fiscal obligations are disciplined by legislation and in particular by the fiscal responsibility law–Complementary Law No. 101^5^^,^ 8.

Interestingly, before Complementary Law No. 101, there was no legislation that guided public indebtedness, spending limits and contracting of credit operations, which provided a real chaos in the budgets of government entities, causing the accumulation of immense, poorly planned debts that went from mandate to mandate^5^^,^ 7.

The absence of legislation such as this one was and has been up to nowadays the biggest cause of the existence of unfinished works and indebted public entities.

The fiscal responsibility law came to establish parameters related to the public spending of each federative entity. The implementation of budget restrictions proposes the fiscal preservation of entities, in accordance with their annual balance sheets, ensuring the financial health of states and municipalities^5^.

## Funding

The main source of private funding, financing is the most common alternative for supplementing budgets and making major investments.

In Brazil, the main financiers are the development banks, which are used for both public and private projects of long term and high value.

Briefly, development banks are financial institutions, usually set up by governments, and provide adequate supply of medium and long-term financing for economic and social development programs and projects.

Currently, the main lenders, which account for 50% of the Brazilian banking system, are Caixa Econômica Federal, Banco do Brasil and Banco Nacional de Desenvolvimento Econômico e Social. In addition to these, some national banks also act as capital providers for long-term financing^2^^,^ 6.

For the most part, financing is desired to expand and/or modernize the physical structures of health institutions, to improve and increase the capacity of care and the provision of health services, but it still meets demands for modernization of management, governance, operational efficiency, and certification, aiming at economic and financial sustainability.

## Public-private partnership and expression of interest procedure

Inserted in the national scenario by Federal Law No. 11,079/2003, public private partnerships are commonly presented as the union of a group of actors with a common goal of improving health, with mutually agreed roles and principles^7^. They consist of three factors:

- At least one private non-profit organization and one for-profit organization;
- Sharing of efforts and benefits;
- Creating social values to improve health.

Subject to the rules imposed by government regulators, they can come up with breakthroughs and innovative solutions to provide additional resources for public health research.

The expression of interest procedure allows individuals or legal entities to present feasibility studies for a specific project, subsidizing with information suitable for the opening of a concession or public private partnership process.

The winning study that will be used for the implementation of the project will have the reimbursement of the expenses of the elaboration of the project, while the other ones do not have any valid reimbursement.

Expression of interest procedure can be applied in public private partnership projects, concession, permission and lease of services and public goods, concession of real right of use, privatization of companies and partnership contracts.

## Public notices

Public notices are public calls opened by legal entities, which, through the proposed criteria, select normative Manager procedures to receive resources or awards provided for in the notice.

It is a very important source of fundraising, being recommended for all types of organization, as in 2019 the number of calls for proposals moved more than R$ 3 billion^11^.

They are extremely democratic and are made available throughout the year, for the most diverse causes, for the most diverse stakeholders, and at flexible values adaptable to demands and projects.

The competition is broad and requires that the projects are well prepared and planned, meeting the proposed needs, and the criteria required in the notices. Upon approval, the resource must be used exclusively for the purpose for which it was registered and estimated in the work plan^15^.

## Final considerations

Finally, fundraising, regardless of whether it is public or private, is an important step and should be discussed by the researchers involved. It is important that this fundraising is strategic, structured, with clear objectives and that it considers all the financial needs involved, such as administrative expenses, human resources, and maintenance of the physical structure.
